# Ectopic migration of the mandibular third molar to the condylar region: A narrative bibliographic review of a case

**DOI:** 10.4317/jced.63224

**Published:** 2025-10-17

**Authors:** María Lacalzada-Pastor

**Affiliations:** 1 Master’s Degree in Periodontics and Oral Implantology. University of Barcelona. Master’s Degree in Oral Medicine. University of Barcelona

## Abstract

**Background:**

Dental inclusions are relatively common; however, inclusions in the condylar, subcondylar, or coronoid process regions are extremely rare.

**Case description:**

This paper describes a clinical case of ectopic migration of a third molar to the condylar region of the right mandibular ramus. To update knowledge about this entity, its possible etiology, clinical characteristics, and therapeutic options, a review of the literature from the last 25 years, from 2000 to 2025, was conducted.

**Conclusions:**

Ectopic migration of mandibular third molars to the condylar region is a rare condition. In most cases, it causes pathology that must be treated. Therapeutic treatment should be based on symptoms, radiological findings, and the functional status of the temporomandibular joint. In the absence of symptoms and pathology, conservative treatment with follow-up may be a safe and appropriate option, as in the case presented.

## Introduction

The third molar is, without doubt, the most frequently impacted tooth. The prevalence of impacted mandibular third molars is 20% to 30%, with a higher prevalence in women (1). Although the third molar is frequently impacted, its location in an ectopic position displaced from its normal anatomical location is extremely rare (2), and in the condylar or subcondylar region even more so, so knowledge about its etiology is limited (1 , 2). This paper aims to report the case of an ectopic mandibular third molar in the mandibular condyle of a 43-year-old female patient and to update knowledge about this entity, its possible etiology, clinical characteristics, and therapeutic options, by conducting a database search of the last 25 years. Given its low historical prevalence, the etiology of tooth migration remains unclear (1 , 3). There are several theories that attempt to explain the ectopic position of some third molars. Cases of third molar migration associated with dentigerous cysts have been described, arguing that these cysts produce fluid that can compress and displace the molars. This displacement could also be caused by trauma or ectopic formation of the cysts (3). Other theories support that it may be associated with mandibular disorders, pathological conditions, or iatrogenesis (2). Clinical manifestations vary in each case; the most prevalent are pain, facial swelling, and trismus; other times, cutaneous fistulas may occur or the patient may be asymptomatic. The approach to managing the case will depend primarily on the symptoms it causes and the associated pathology. In this study, we found cases resolved with surgical intervention, either intraorally (endoscopic or not) or extraorally. If the findings are incidental and without associated pathology, another valid option is to agree with the patient on a wait-and-see approach with periodic follow-ups, as was the case presented in this review.

## Case Report

**Figure 1:**
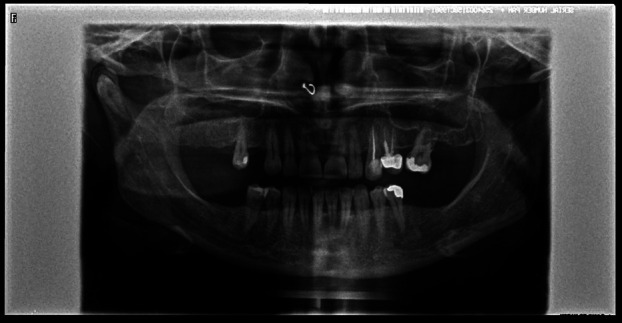
Orthopantomography.

The patient is a 43-year-old woman who presented with a routine checkup. She presented no symptoms except for occasional hypersensitivity. Her medical history revealed no significant medical history, except for a hiatal hernia and liver inflammation of more than 20 years standing. She is taking no medication and reports no surgical interventions or toxic habits. She also has no known allergies. Regarding her dental history, she requires conservative treatment (fillings and root canals) and is partially edentulous, thus wearing two removable prostheses. An orthopantomography revealed an impacted tooth in the condylar region as a chance finding (Fig. 1).

1

**Figure 2:**
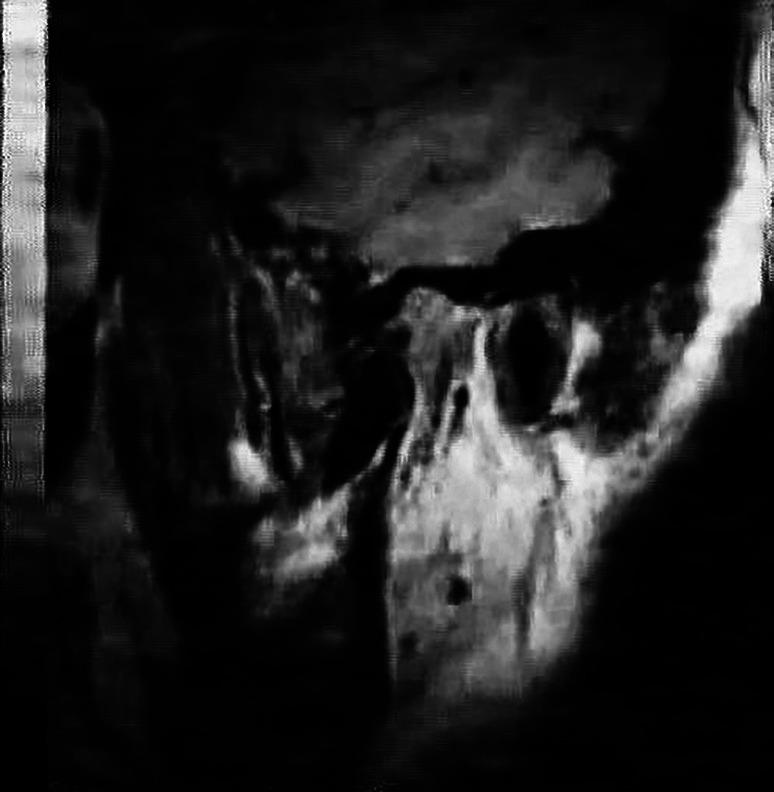
Right TMJ MRI.

**Figure 3:**
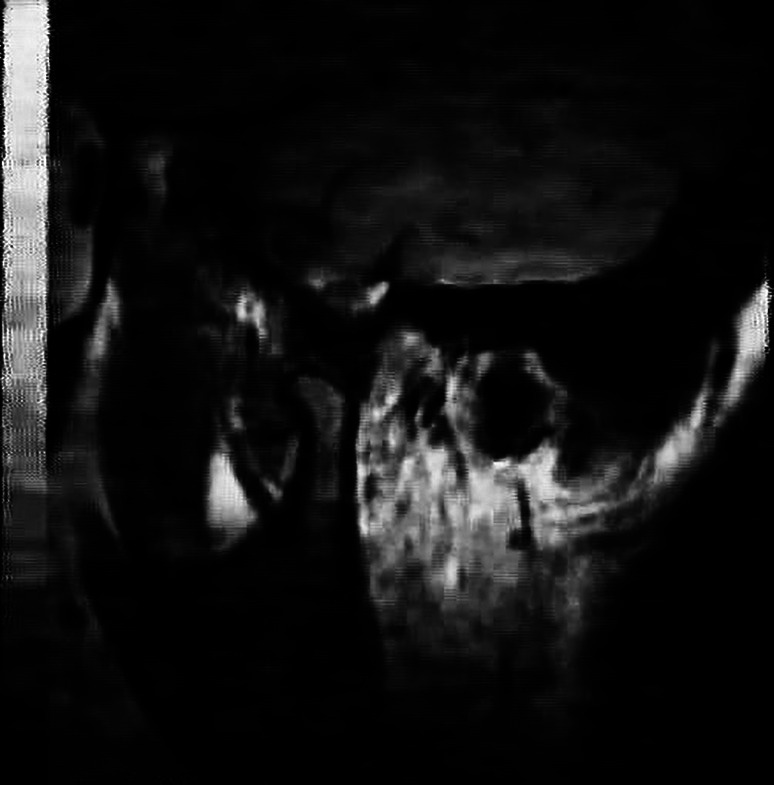
Left TMJ MRI.

The patient confirmed that this anomaly was observed nine years ago on another routine panoramic radiograph, and an MRI was requested (Figs. 2,3).

2

3

The MRI clearly shows the inclusion of a tooth in the right condyle, with intact cortical bone and no deformities. The joint structures are congruent, and the meniscus is correctly positioned, maintaining functionality. The patient has no associated symptoms and has never reported trismus or temporomandibular joint dysfunction. Joint examination revealed no noises or limited centric or eccentric movements. Muscle palpation was unremarkable.

## Discussion and Conclusions

The incidence of ectopic third molars in the condylar region is extremely low. From 2000 to 2025, only 17 cases were reported, 20 since 1980, most of which were found in women. Some of these cases are diagnosed because they cause infection or pain, but others are asymptomatic and, like the case reported here, are incidental findings. The most useful imaging techniques for diagnosis are panoramic radiographs, CT, and MRI. Panoramic radiographs identify the presence of the ectopic molar, while CT accurately assesses its three-dimensional location, and MRI provides information on soft tissue structures and the condition of the TMJ. The locations of the ectopic migrations were as follows: the mandibular ramus (1 , 11), the condyle [4, 3A, 6A, 6B, 7, 8, 15, 16, and the present case], and the subcondylar region [2, 3B, 5, 10, 13, 14, and 17]. Many of the inclusions were associated with cysts, 14 of which were recorded in the review by Iglesias-Martín (2) and in the case by Wang (1) and Bowman (17), one with radiolucency not histologically confirmed (10), as in the present case, and another without clear evidence of an associated cyst (15). In the Iglesias-Martín review from 1980 to 2012, they identified 2 articles on impacted third molars in the mandibular ramus, 10 articles on the coronoid process, and 14 articles on the condylar/subcondylar region. A total of 14 well-documented cases were included in the literature, plus their own case (2). Nine women and six men, their case being a woman. This review also found the case of Wang et al. in 2007, a 31-year-old woman with an ectopic molar located in the upper part of the ascending ramus, which caused pain and swelling, so they decided to operate on her using an intraoral approach (1). From 2012 to the present, four more cases have been reported, including the one presented in this review. Three cases were women and two were men. The Bowman case was reported in 2014, the Okuyama et al. case was reported in 2017, the Liau et al. case was reported in 2017, the Campbell et al. case was reported in 2020, and the one published in this review in 2025. This study includes a total of 21 cases, 20 more than the one presented here, (Table 1).

Table 1

This table is based on the previous review by Iglesias-Martín et al. (2012), updated with more recent studies identified in this search. Several theories have been proposed to explain the ectopic position of third molars, including aberrant eruption, trauma, and ectopic tooth bud formation (2 , 3). A third molar may be displaced a large distance from its normal position due to incomplete eruption, displacement caused by lesions such as bone cysts or tumors, or altered eruption due to odontogenic tumors (2 , 7). Most reported cases were associated with radiolucent lesions on panoramic radiographs and were histopathologically confirmed as dentigerous cysts (2). Therefore, the theory involving odontogenic cysts in the pathogenesis of ectopic third molars appears to be the most compelling (3). However, it cannot be affirmed as the sole cause, as there are other cases without associated cystic pathology [10, 15, 17 and the one reported in this review]. The most common symptoms associated with an ectopic third molar are pain and swelling in the ipsilateral mandible or preauricular region, trismus, difficulty chewing, and temporomandibular joint dysfunction. Pain in the masticatory muscles or fever due to inflammation may also occur (1). Most cases cause symptoms such as pain [1-8,10,11,13,15, and 16], trismus [3,10,2, and 15], facial swelling [1-8,10,11 and 15-17], or fistulas [13 and 16]. Others are asymptomatic, as in the case described here. Treatment may be conservative in asymptomatic cases or surgical with an intraoral or extraoral approach in the presence of complications or associated pathology [1-8, 4, 10, 11, 13-15 and 17]. There is a higher prevalence in women [1, 2, 3A, 6A, 7, 8, 11, 15 and 16] with an average age of 50.65 years (the oldest patient being 68 years old and the youngest being 30). To establish a treatment plan, it is necessary to assess each case individually, including the morbidity caused by the ectopic tooth and its association, if any, with a dentigerous cyst, temporomandibular dysfunction, and the high potential risk of condylar fracture. Ectopic molars should be removed if they cause severe symptoms or are associated with cystic pathology, always taking into account case variability and the trauma caused by surgery and its potential complications, such as fractures. The surgical approach should be planned according to the location and position of the ectopic third molar, always considering the potential morbidity associated with surgery (9). There are several guidelines and surgical techniques used in cases of tooth migration to the condylar region. Several are described in the literature and, depending on the approach, are classified as: preauricular, retromandibular, intraoral, and endoscopic (3). The most conservative surgical technique is the one that uses endoscopic access (4). 100% of the patients operated on in this review presented symptoms and/or associated pathology [1-8, 10, 11, 13-15, 16, and 17]. The final decision on the treatment plan will depend largely on the pathology associated with the tooth impaction, the patient's cooperation, the surgeon's skill, and their experience with the techniques used. Several surgical techniques are described in the literature, which are classified according to the approach route as: preauricular, retromandibular, intraoral, and endoscopic (3). In the cases reported in the literature, some authors opt for an extraoral approach (3 , 5 , 10 , 13 , 17), while others prefer an intraoral approach (1 , 6 , 7 , 11 , 14 - 16). The endoscopic technique is sometimes the technique of choice (4 , 14). In the present case, given the absence of symptoms and the stability of the pathology over the years, it was decided, in conjunction with the patient, to implement expectant management and annual radiographic follow-ups.

## Figures and Tables

**Table 1:** Table adapted from Iglesias-Martín et al. (2012), with additions from the literature between 2012 and 2025 and the case reported by Wang et al. in 2007.

Authors/year	Age	Gender	3th molar position	Symptoms and signs	Treatment
Burton y Scheffer. 1980	57	F	R y C (bilateral)	Left side: swelling and pain. Right side: no symptoms.	Left side: intraoral SRRight side: extraoral SR
Srivastava y Singh. 1982	40	F	C	Discharging preauricular fistula	Conservative
Bux y Lisco. 1994	66	F	SC	Pain, swelling, trismus and fistula	Extraoral SR
Medici et al. 2001	41	F	C	Pain and swelling	Intraoral SR
TÃ¼mer et al. 2002	47	M	SC	Pain and swelling	Extraoral SR
Wassouf et al. 2003	49		C	Pain and swelling	Intraoral SR. Reconstruction with bone chips from iliac crest
Suarez-Cunqueiro et al. 2003	45	M	C	Pain and swelling	Intraoral SR (endoscopically assisted)
Salmeron et al. 2007	53	M	C	Pain, swelling and trismus	Extraoral SR
Salmeron et al. 2007	42	M	SC	Pain and swelling	Extraoral SR
Gadre et Waknis. 2010	30	F	C	Pain and swelling	SR intraoral
Gadre et Waknis. 2010	40	M	C	Pain and swelling	Intraoral SR
Bortoluzzi et Manfro. 2010	68		C	Pain, swelling and fistula	Intraoral SR
Pace et al. 2010	53	M	SC	Pain, swelling and fistula	Extraoral SR. Fixation with 1 miniplate
Shivashankara et al. 2011	45	M	SC	Pain, swelling and trismus	Extraoral SR
Iglesias-MartÃ­n et al. 2011	53	F	SC	Pain, swelling and trismus	Extraoral SR. Fixation with 1 miniplate
Wang et al. 2007	31	F	R	Pain and swelling on the right side	Intraoral access
Bowman et al. 2014	56	M	SC	Diffuse preauricular swelling	Extraoral approach (anteroparotid transmasseteric)
Okuyama et al. 2017	63	F	R	Right preauricular facial swelling, mandibular functional limitation and pain	Intraoral approach to the medial aspect of the ramus
Liau et al. 2017	X	M	SC	Acute infection of the fascial space	Endoscopically assisted intraoral approach
Campbell et al. 2020	60	F	R/C	Left facial edema, pain and trismus	Intraoral approach SR
Case reported by the author 2025	43	F	C	Asymptomatic	Expectant management

M: male; F: female; R: mandibular ramus; C: condylar region; SC: subcondylar region; SR: surgical removal

## Data Availability

The datasets used and/or analyzed during the current study are available from the corresponding author.
